# Correlation Between CT Features of Active Tuberculosis and Residual Metabolic Activity on End-of-Treatment FDG PET/CT in Patients Treated for Pulmonary Tuberculosis

**DOI:** 10.3389/fmed.2022.791653

**Published:** 2022-02-22

**Authors:** Ismaheel O. Lawal, Kgomotso M. G. Mokoala, Matsontso Mathebula, Ingrid Moagi, Gbenga O. Popoola, Nontando Moeketsi, Maphoshane Nchabeleng, Chris Hikuam, Jerrold J. Ellner, Mark Hatherill, Bernard P. Fourie, Mike M. Sathekge

**Affiliations:** ^1^Department of Nuclear Medicine, University of Pretoria, Pretoria, South Africa; ^2^Nuclear Medicine Research Infrastructure, Steve Biko Academic Hospital, Pretoria, South Africa; ^3^Department of Medical Microbiology and MeCRU, Sefako Makgatho University of Medical Science, Pretoria, South Africa; ^4^Department of Epidemiology and Community Health, University of Ilorin, Ilorin, Nigeria; ^5^South African Tuberculosis Vaccine Initiative, Department of Pathology, Institute of Infectious Disease and Division of Immunology, University of Cape Town, Cape Town, South Africa; ^6^Department of Medicine, Centre for Emerging Pathogens, Rutgers-New Jersey Medical School, Newark, NJ, United States; ^7^Department of Medical Microbiology, University of Pretoria, Pretoria, South Africa

**Keywords:** pulmonary tuberculosis, end-of-treatment, FDG PET/CT, residual metabolic activity, computed tomography, HIV infection

## Abstract

Patients who complete a standard course of anti-tuberculous treatment (ATT) for pulmonary tuberculosis and are declared cured according to the current standard of care commonly have residual metabolic activity (RMA) in their lungs on fluorine-18 fluorodeoxyglucose positron emission tomography/computed tomography (FDG PER/CT) imaging. RMA seen in this setting has been shown to be associated with relapse of tuberculosis. The routine clinical use of FDG PET/CT imaging for treatment response assessment in tuberculosis is hindered by cost and availability. CT is a more readily available imaging modality. We sought to determine the association between CT features suggestive of active tuberculosis and RMA on FDG PET/CT obtained in patients who completed a standard course of ATT for pulmonary tuberculosis. We prospectively recruited patients who completed a standard course of ATT and declared cured based on negative sputum culture. All patients had FDG PET/CT within 2 weeks of completing ATT. We determined the presence of RMA on FDG PET images. Among the various lung changes seen on CT, we considered the presence of lung nodule, consolidation, micronodules in tree-in-bud pattern, FDG-avid chest nodes, and pleural effusion as suggestive of active tuberculosis. We determine the association between the presence of RMA on FDG PET and the CT features of active tuberculosis. We include 75 patients with a mean age of 36.09 ± 10.49 years. Forty-one patients (54.67%) had RMA on their FDG PET/CT while 34 patients (45.33%) achieved complete metabolic response to ATT. There was a significant association between four of the five CT features of active disease, *p* < 0.05 in all cases. Pleural effusion (seen in two patients) was the only CT feature of active disease without a significant association with the presence of RMA. This suggests that CT may be used in lieu of FDG PET/CT for treatment response assessment of pulmonary tuberculosis.

## Introduction

Pulmonary tuberculosis (PTB), caused by drug-sensitive strains of *Mycobacterium tuberculosis* (DS-TB), is treated with multiple drugs for 6 months (1). This long duration of treatment with multiple drugs has a cost implication and a risk of non-adherence predisposing to the development of drug resistance. In recent times, many trials have been done to guide the design of shorter treatment regimens for PTB (2–5). A reliable biomarker of cure is a crucial tool for the determination of sterilizing cure of tuberculous disease. None of the currently utilized biomarkers is sufficiently reliable in predicting cure. The current gold-standard technique for the assessment of successful treatment of tuberculous disease is sputum culture. Sputum culture may be negative despite persisting bacilli. A reason for negative culture in the presence of live bacilli may be due to the slow-growing nature of these bacilli that makes them non-culturable (6). Therefore, other biomarkers that can reliably predict sterilizing cure of tuberculosis are needed for the rational design of a shorter treatment course of ATT.

Metabolic imaging with fluorine-18 fluorodeoxyglucose (FDG) using hybrid positron emission tomography/computed tomography (PET/CT) scanners has been evaluated for their utility as a non-invasive tool for therapy response assessment in patients with tuberculosis. Findings on FDG PET/CT obtained at baseline can reliably discriminate between responders vs. non-responders to anti-tuberculous therapy (ATT) (7, 8). Similarly, FDG PET/CT obtained as early as 1 month into ATT can predict successful treatment outcome (9). More importantly, our group and others have recently shown that patients with residual metabolic activity (RMA) in their lungs on FDG PET/CT obtained at treatment completion have a higher risk of TB relapse (10, 11). Conversely, patients who achieved complete metabolic response to ATT at end-of-treatment (EOT) FDG PET/CT have a durable cure with no risk of TB relapse (11). These studies support the utility of FDG PET/CT as a useful non-invasive tool for treatment response assessment in patients treated for tuberculosis.

Despite its excellent performance as a tool for treatment response assessment, the application of FDG PET/CT is unlikely to become routine in clinical practice due to cost and availability issues. The availability and utilization of molecular imaging techniques are still very limited in developing countries where most patients with tuberculosis live (12, 13). Computed tomography (CT) is a more widely available imaging tool in TB-endemic regions of the world. Certain features on CT, including lung consolidation, lung nodules in the typical tree-in-bud pattern, pleural effusion, and others, are known to suggest active PTB (14–16). Determining the strength of the association between these CT features suggestive of active TB and the RMA on FDG PET is crucial for guiding the application of CT, a more widely available imaging technique, for treatment response assessment of patients treated for PTB. Therefore, in this study, we aimed to determine the association between RMA on FDG PET and CT features of active disease in patients treated with a standard regimen of anti-tuberculous medication.

## Methods

### Patients

Between December 2017 and December 2019, we prospectively recruited patients aged 18 years and above who completed a 6-month regimen of ATT consisting of a standard dose of isoniazid, rifampicin, pyrazinamide, and ethambutol for 2 months followed by rifampicin and isoniazid for 4 months (17). In all participants, the diagnosis of DS-PTB was by positive Gene-Xpert MTB/RIF (Cepheid) and culture-confirmed sensitivity to all first-line agents. All patients completed ATT and were declared cured based on a negative culture. Exclusion criteria were unknown human immunodeficiency virus (HIV) status, previous history of tuberculous disease within 3 years prior to the index case, presence of symptoms suggestive of TB after treatment, identification of tubercle bacilli on GeneXpert MTB/RIF (Cepheid) or culture after ATT, receipt of non-standard anti-tuberculous drug regimen or the use of investigative agents or vaccine, ongoing use of immunosuppressive drugs including steroids, concurrent illness or treatment other than HIV infection and its treatment, uncontrolled diabetes mellitus, pregnancy or breastfeeding status, anemia defined as a hemoglobin level below 7g/dL, and significant smoking history defined as >30 pack-year. Details regarding patient recruitment, the comprehensive assessment at the end of ATT, patient preparation for FDG PET/CT, and the details regarding FDG PET/CT acquisition have been previously published (11). This study was approved by the Research Ethics Committee of the Faculty of Health Sciences University of Pretoria, Pretoria, South Africa and Sefako Makgatho Health Sciences University, Pretoria, South Africa. All participants provided written informed consent prior to inclusion in this study.

### FDG PET/CT Image Analysis

Two experienced nuclear medicine physicians (IOL and KMGM with 8- and 10-years' experience reading FDG PET/CT scans, respectively) performed FDG PET/CT analysis independently on a dedicated workstation equipped with a syngo.via software (Siemens Medical Solution, Chicago, IL, USA). Areas of disagreement were resolved by consensus. FDG PET and CT images were interpreted independently. On the FDG PET images, we performed a qualitative assessment for significant RMA within the lungs of the patients. We defined significant RMA as residual FDG avidity within the lung tissue that is above background mediastinal activity. We assessed for the presence of features of active tuberculosis vs. residual lung damage due to TB in the lungs of the patients. We considered the following lesions as suggestive of active tuberculosis—lung nodules, lung consolidation, micronodules in the tree-in-bud pattern, FDG-avid thoracic lymph nodes, and pleural effusion. We considered cystic lung changes, cavitary pulmonary lesions, bronchiectetic changes, calcification, and fibrotic lung changes as suggestive of inactive result lung changes to previous PTB disease.

### Statistical Analysis

We present qualitative data as frequency (%) and quantitative variables as mean ± standard deviation if normally distributed or median (interquartile range, IQR/range) if skewed. We used the Chi-square test to compare the differences in the variables of patients who achieved complete metabolic response (CMR) to ATT compared with patients who had RMA at the end of treatment for pulmonary tuberculosis. We also used the Chi-square test to compare the frequency of the CT features of active and inactive disease in patients who achieved CMR vs. patients who had RMA at the end of ATT. We performed a logistic regression to determine the strength of association between CT features suggestive of active TB disease and RMA. Using forward logistic regression analysis, we evaluated the patient-related factors and CT findings predictive of RMA on FDG PET/CT at the end of ATT. We set statistical significance at a *P*-value < 0.05. We performed statistical analysis using SPSS statistics version 21.0 (IBM Corp., Armonk New York, USA).

## Results

### Patients

In total, 75 patients who completed a standard course of ATT were recruited to undergo an EOT FDG PET/CT. There were 47 (62.67%) males. The mean age of the study population was 36.09 ± 10.49 years. There were 50 HIV-infected patients with a median CD4 T cell count of 255 (IQR = 147–448) cells/μL. All the 50 HIV-infected patients were on antiretroviral therapy (ART) with a completely suppressed HIV viremia achieved in 35 (70.00%) patients while 15 patients had a detectable HIV viremia (median HIV viral load = 12, 497 copies/mL) at the time of EOT FDG PET/CT scan. The detailed clinical and demographic information of the patients are presented in Table 1.

**Table 1:** Demographic and clinical characteristics of the study participants.

**Variable**	**Frequency**	**Percent**
**Age**		
<45	60	80.0
≥45	15	20.0
Mean ± SD	36.09 ± 10.49
Range	20–65
**Gender**		
Male	47	62.7
Female	28	37.3
**HIV**
Yes	50	66.7
No	25	33.3
**CD4 T cell count (cells/μL)**		
Median (IQR)	255 (147–448)
**HIV viral load (copies/mL)**		
Detectable	15	30.0
Not detectable	35	70.0
**Viral load (detectable)**		
Median (IQR)	12,497.00 (158.00–38,841.00)
**Smoking**		
Yes	25	33.3
No	50	66.7
**BMI (kg/m** ^**2**^ **)**		
Mean ± SD	21.88 ± 5.90
Range	15.19–48.15
**Hemoglobin (g/dL)**		
Mean ± SD	13.41 ± 1.93
Range	8.80–17.00
**CRP (mg/L)**		
Median (IQR)	4.31 (1.36–11.06)

*HIV, Human Immunodeficiency Virus; CD4, Cluster of Differentiation 4; BMI, Body Mass Index; CRP, C-Reactive Protein*.

### Findings on End-of-Treatment FDG PET/CT Imaging

All patients completed an EOT FDG PET/CT. Forty-one patients (54.67%) had RMA on EOT FDG PET/CT while 34 patients (45.33%) achieved CMR to ATT. The most prevalent lung lesions seen on the CT images were fibrotic lung changes, lung nodules, bronchiectetic changes, and cavitary lung changes seen in 55, 53, 35, and 30 patients, respectively. The rarest CT lung changes seen were lung calcification seen in five patients and pleural effusion seen in 2 patients. Most patients had more than one type of CT change in their lungs, with 59 patients (78.67%) having bilateral lung CT changes on their EOT FDG PET/CT scans. Table 2 shows the detailed findings on the FDG and CT components of the EOT scan of the patients.

**Table 2:** Summary of findings on end-of-treatment FDG PET/CT scans.

**Variable**	**Frequency**	**Percent**
**Residual metabolic activity**		
Yes	41	54.7
No	34	45.3
**CT findings**		
Bilateral lung changes	59	78.7
Cavitary lung changes	30	40.0
Cystic lung changes	14	18.7
Nodules	53	70.7
Consolidation	10	13.3
Bronchiectasis	35	46.7
Calcification	5	6.7
Fibrotic changes	55	73.3
Tree-in-bud pattern	29	38.7
Mediastinal/hilar nodes	17	22.7
Pleural effusion	2	2.7

### Association Between the Presence of RMA on FDG PET and Patients' Characteristics

We evaluated the association between the presence of RMA on EOT FDG PET/CT vs. specific clinical and demographic characteristics of the study population. Patients with RMA on their EOT FDG PET/CT were significantly more likely to be older than 45 years, have a CD4 T cell count of above 200 cells/μL, and an undetectable HIV viremia if HIV-infected. The presence of HIV infection, by itself, was not significantly associated with the presence of RMA on EOT FDG PET/CT. Similarly, other variables like the hemoglobulin level, serum level of C-reactive protein (CRP), patient's gender, and body mass index (BMI) were not significantly associated with the presence of RMA on EOT FDG PET/CT (Table 3).

**Table 3:** Association between residual metabolic activity and patients' characteristics.

	**Residual metabolic activity**		
	**Yes**	**No**	**Total**	**OR (95% CI)**	**χ^2^**	***P*** **-value**
**Variable**	***N*** **= 41(%)**	***N*** **= 34(%)**	***N***			
**Age**						
<45	29 (48.3)	31 (51.7)	60	0.234 (0.060–0.914)	4.856	0.028*
≥45	12 (80.0)	3 (20.0)	15			
**Gender**						
Male	25 (53.2)	22 (46.8)	47	0.852 (0.332–2.187)	0.111	0.740
Female	16 (57.1)	12 (42.9)	28			
**BMI (kg/m** ^**2**^ **)**						
<18.5	15 (62.5)	9 (37.5)	24	1.603 (0.594–4.322)	0.874	0.350
≥18.5	26 (51.0)	25 (49.0)	51			
**Hb level (g/dL)**						
<12	12 (75.0)	4 (25.0)	16	2.889 (0.825–10.116)	2.894	0.089
≥12	27 (50.9)	26 (49.1)	53			
**CRP level (mg/dL)**						
<10	26 (53.1)	23 (46.9)	49	0.609 (0.207–1.786)	0.824	0.364
≥10	13 (65.0)	7 (35.0)	20			
**HIV**
Yes	24 (48.0)	26 (52.0)	50	0.434 (0.159–1.189)	2.690	0.101
No	17 (68.0)	8 (32.0)	25			
**CD4 T cell count (cells/μL)**						
<200	4 (20.0)	16 (80.0)	20	0.139 (0.036–0.531)	9.216	0.002*
≥200	18 (64.3)	10 (35.7)	28			
**HIV viral load (copies/mL)**						
Detectable	3 (20.0)	12 (80.0)	15	0.167 (0.040–0.700)	6.731	0.009*
Not detectable	21 (60.0)	14 (40.0)	35			

*χ^2^, Chi square test; OR, Odds ratio; 95% CI, 95% Confidence Interval*;

* *p-value < 0.05; BMI, Body Mass Index; Hb, Hemoglobin; CRP, C-Reactive Protein; CD4, Cluster of Differentiation; HIV, Human Immunodeficiency Virus Infection*.

### Association Between the Presence of RMA on FDG PET and Lung Changes on CT

**Figure 1:**
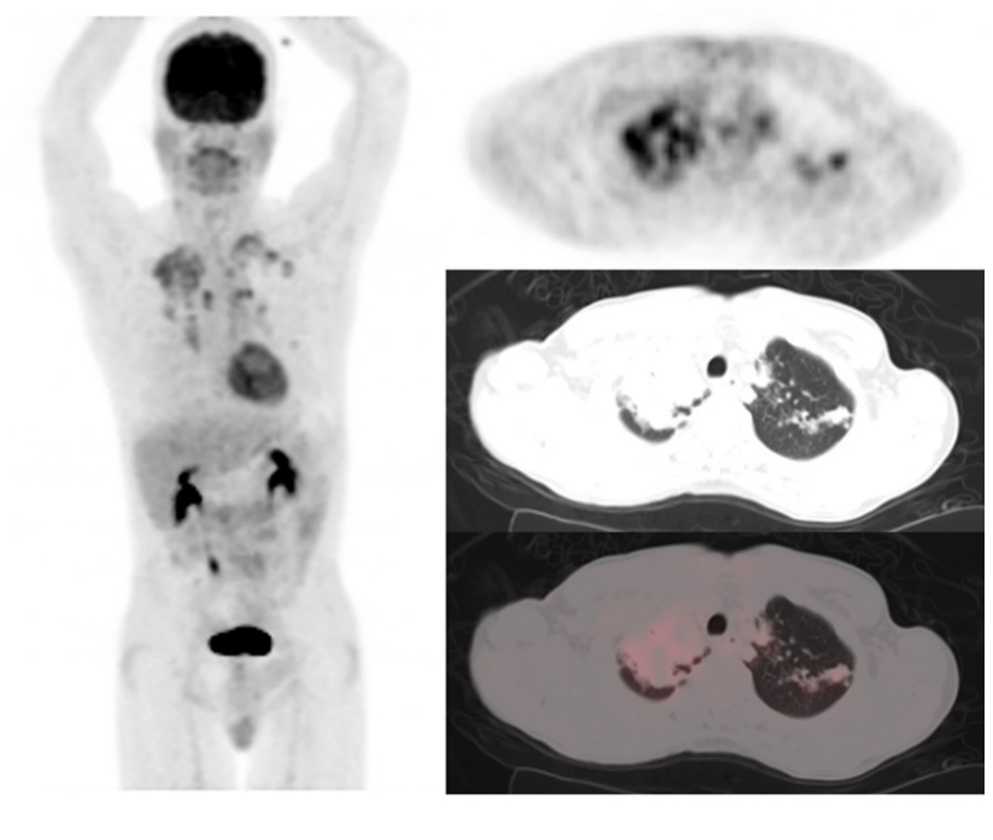
A 49-year-old HIV-infected male who completed a 6-month course of anti-tuberculous treatment. FDG PET/CT obtained for treatment response assessment. The maximum intensity projection scan **(MIPS)** on the left shows residual metabolic activity in the lungs bilaterally, mostly in the upper lobes, right more than left. The left column of images are the PET, CT, and fused PET/CT transverse section through the upper lobes of the lungs showing consolidation of right upper lobe parenchyma and left upper lobe lung nodules all showing residual metabolic activity post-treatment for pulmonary tuberculosis.

**Figure 2:**
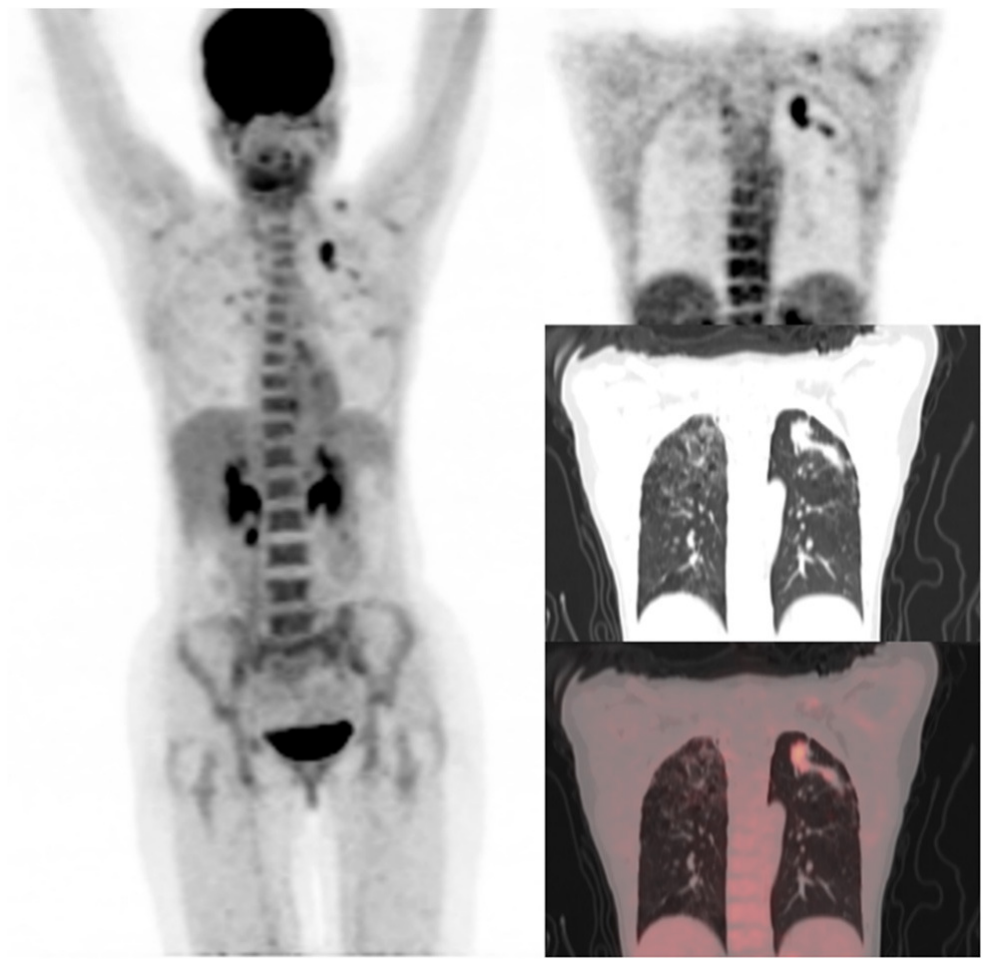
A 31-year-old HIV-infected female who completed a six-month course of anti-tuberculous medication for drug-sensitive pulmonary tuberculosis. Maximum intensity projection scan **(MIPS)** on the left shows a focal area of residual metabolic activity in the left upper lobe. This focus of residual metabolic activity is confirmed to be in left upper lobe lung nodules on the coronal section of PET, CT, and fused PET/CT images shown on the right. Fibrocavitory changes seen in the lungs bilaterally do not demonstrate significant residual metabolic activity.

**Figure 3:**
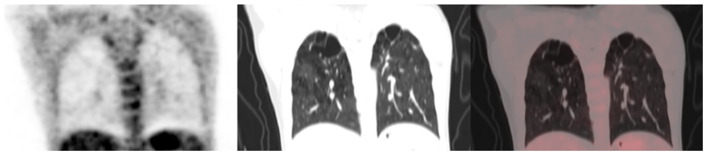
A 40-year-old female status post-treatment for drug-sensitive pulmonary tuberculosis. She is HIV negative. From left to right, coronal section of the PET, CT, and fused PET/CT images are shown. Bilateral fibrocavitory changes seen in the upper lobes show no significant residual metabolic activity.

**Figure 4:**
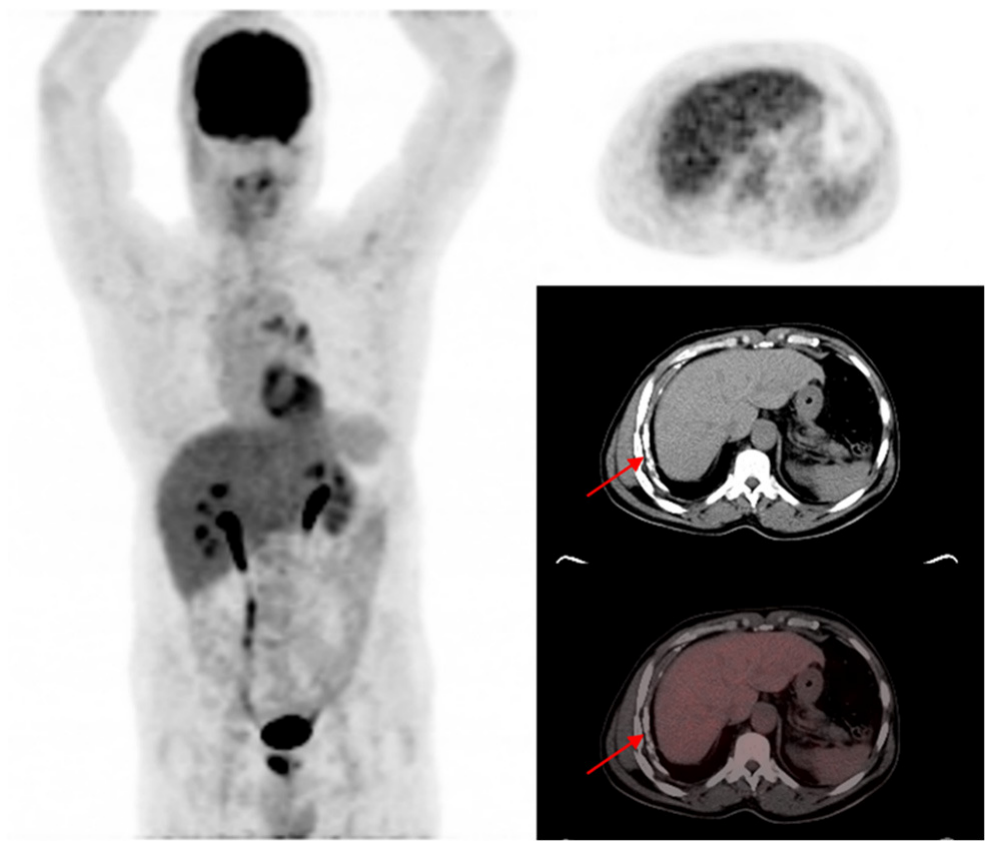
A 58-year-old HIV-infected male who completed a six-month course of anti-tuberculous medication for drug-sensitive pulmonary tuberculosis. The maximum intensity projection scan **(MIPS)** shown on the left does not demonstrate significant residual metabolic activity in the lung fields bilaterally. On the axial PET, CT, and fused PET/CT images shown on the right, pleural calcifications (red arrows) without corresponding significant residual metabolic activity is shown. Calcifications developed in this patient on the background of pleural effusion that complicated the active phase of the tuberculous disease.

**Figure 5:**
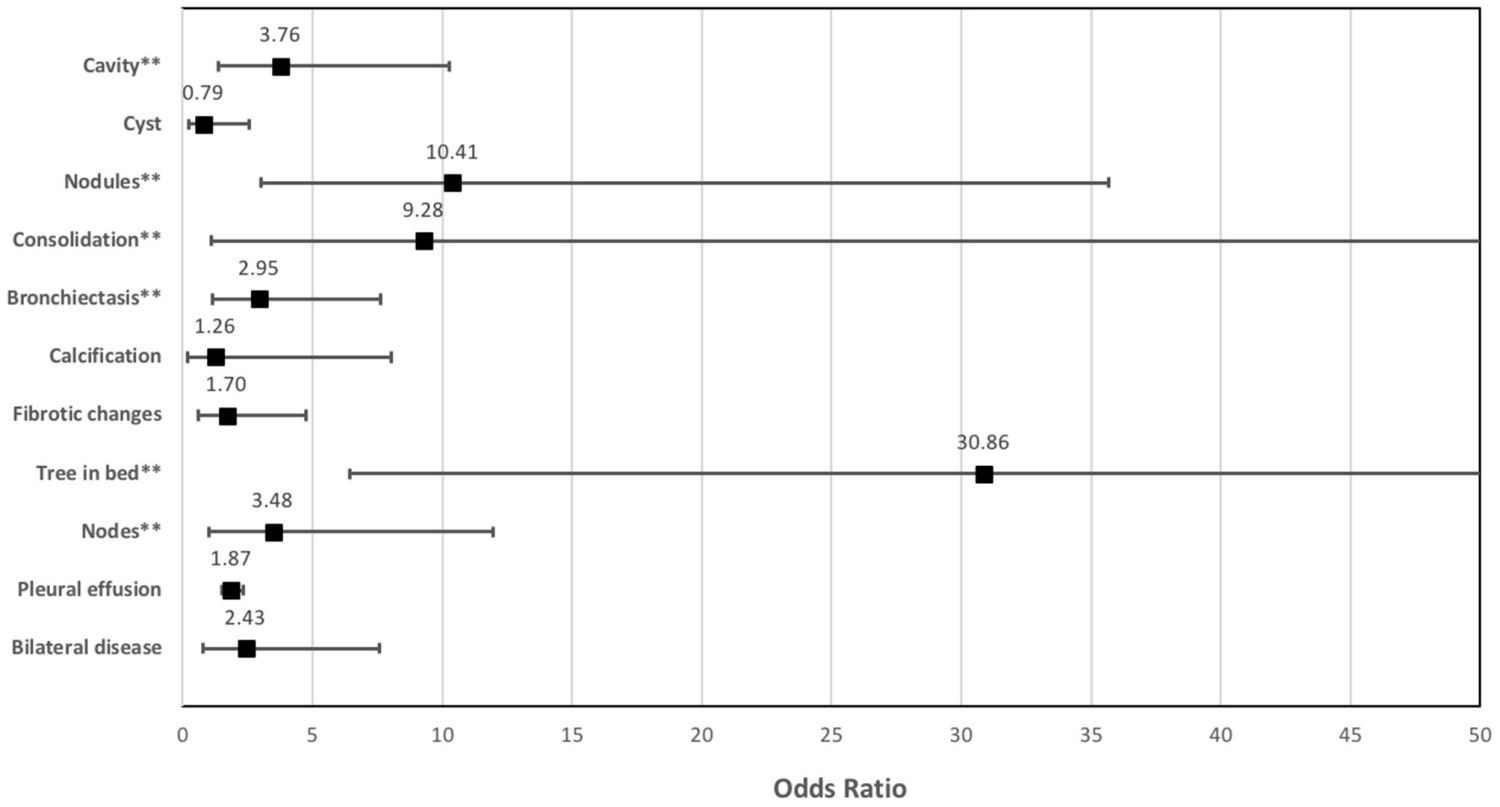
The strength of the association between the different lung changes on CT and the presence of residual metabolic activity on end-of-treatment FDG PET/CT in patients who completed a standard course of anti-tuberculous treatment for pulmonary tuberculosis.

Out of the five CT lung changes we classified as suggestive of active residual tuberculosis, four showed a significant association with the presence of RMA on EOT FDG PET/CT scans (Figures 1, 2). Pleural effusion was the only CT lung change that we classified as suggestive of active residual tuberculosis whose presence was not significantly associated with RMA on EOT FDG PET/CT as the two patients with pleural effusion did not have RMA in their lung fields. Among the five CT features we categorized as suggestive of inactive lung changes after tuberculous disease, two (cavitary lung changes and bronchiectetic changes) were significantly associated with the presence of RMA while the other three (fibrotic lung changes, calcifications, and cystic lung changes) were not (Figures 3, 4). We further evaluated the combined strength of all CT findings suggestive of active tuberculosis in their association with the presence of RMA. We found a strong level of association for the combined variables than the individual variables on their own (Table 4; Figure 5). When analyzed in combination, the five CT findings that were classified as suggestive of inactive post-tuberculous lung changes were not significantly associated with the presence of RMA on EOT FDG PET.

**Table 4:** Association between residual metabolic activity and CT findings on end-of-treatment FDG PET/CT scans.

	**Residual metabolic activity**		
	**Yes**	**No**	**Total**	**OR (95% CI)**	**χ^2^**	***P*** **-value**
**Variable**	***N*** **= 41(%)**	***N*** **= 34(%)**	***N***			
**Cavity**						
Yes	22 (73.3)	8 (26.7)	30	3.763 (1.381–10.253)	7.030	0.008*
No	19 (42.4)	26 (57.8)	45			
**Cyst**						
Yes	7 (50.0)	7 (50.0)	14	0.794 (0.248–2.541)	0.151	0.697
No	34 (55.7)	27 (44.3)	61			
**Nodules**						
Yes	37 (69.8)	16 (30.2)	53	10.406 (3.036–35.672)	16.722	<0.001*
No	4 (18.2)	18 (82.8)	22			
**Consolidation**						
Yes	9 (90.0)	1 (10.0)	10	9.281 (1.111–77.512)	5.813^F^	0.018*
No	32 (49.2)	33 (50.8)	65			
**Bronchiectasis**						
Yes	24 (68.6)	11 (31.4)	35	2.952 (1.142–7.632)	5.120	0.024*
No	17 (42.5)	23 (57.5)	40			
**Calcification**						
Yes	3 (60.0)	2 (40.0)	5	1.263 (0.199–8.033)	0.061^F^	1.000
No	38 (54.3)	32 (45.7)	70			
**Fibrotic changes**						
Yes	32 (58.2)	23 (41.8)	55	1.700 (0.606–4.768)	1.028	0.311
No	9 (45.0)	11 (55.0)	20			
**Tree-in-bed pattern**						
Yes	27 (93.1)	2 (6.9)	29	30.857 (6.435–147.967)	28.187	<0.001*
No	14 (30.4)	32 (69.6)	46			
**Nodes**						
Yes	13 (76.5)	4 (23.5)	17	3.482 (1.014–11.953)	4.217	0.040*
No	28 (48.3)	30 (51.7)	58			
**Pleural effusion**						
Yes	2 (100.0)	0 (10.0)	2	1.872 (1.511–2.319)	1.704^F^	0.498
No	39 (53.4)	34 (46.6)	73			
**Bilateral disease**						
Yes	35 (59.3)	24 (40.7)	59	2.431 (0.779–7.582	2.419	0.120
No	6 (37.5)	10 (62.5)	16			
**CT findings of inactive disease**						
Yes	35 (59.3)	24 (40.7)	59	2.431 (0.799–7.582)	2.419	0.120
No	6 (37.5)	10 (62.5)	16			
**CT findings of active disease**						
Yes	29 (90.6)	3 (9.4)	32	24.972 (6.392–97.561)	29.121	<0.001*
No	12 (27.9)	31 (72.1)	43			

*χ^2^, Chi square test; OR, Odds ratio; 95% CI, 95% Confidence Interval*;

* *p-value < 0.05; CT, Computed Tomography; F, ANOVA (Analysis of Variance)*.

### Predictors of RMA on End-of-Treatment FDG PET/CT

To determine the effect of all variables (patient-related and CT findings) with significant associations with RMA while controlling for the effect of the individual variable, we performed a binary logistic regression to determine the significant predictors of RMA. Using a forward logistic regression method, we found that only the combined CT features of active tuberculous disease and the presence of lung nodules were significant predictors of RMA on EOT FDG PET/CT (Table 5). Other variables, including age, cavitary lung changes, lung consolidation, bronchiectetic changes, a tree-in-bud pattern of micronodules, mediastinal/hilar lymph nodes, CD 4 count T cell, and HIV viral load, which all showed a significant association with RMA on their own were excluded from our models. In the first step of our binary logistic regression analysis, only the combined CT features of active disease significantly predict RMA with an increase odd of 25.714 (95% CI: 4.704–140.579). Step 2 adjusted for the effect of the combined CT features of active disease and the presence of nodules. Both variables (combined CT features of active tuberculous lung changes and the presence of lung nodules) were found to independently predict RMA (*p*-values 0.002 and 0.016, respectively), Table 5.

**Table 5:** Predictors of RMA on EOT FDG PET/CT.

	**Step 1**			**Step 2**		
**Variables**	**B**	***P*** **-value**	**OR (95% CI)**	**B**	***P*** **-value**	**OR (95% CI)**
**CT features of active TB**						
Yes	3.247	<0.001*	25.714(4.704–140.579)	3.630	0.002*	37.701(4.001–355.239)
No ^REF^			1			
**Nodules**	–	–	–			
Yes				2.937	0.016*	18.855(1.731–205.435)
No ^REF^						

*Method: Forward Logistic Regression. B: Coefficient of Binary Logistic Regression; OR, Odds ratio; 95% CI, 95% Confidence interval; REF, Reference category*;

* *p-value < 0.05; TB, Tuberculosis. **Step 1**_ R^2^: 0.470. Predictive Value: 81.3%, χ^2^: 20.775, p < 0.001. **Step 2**_ R^2^: 0.619. Predictive Value: 81.3%. χ^2^: 29.878, p < 0.001. **Variables excluded**: Age, Cavitatory lung changes, Lung consolidation, Bronchiectasis, Tree-in-bud pattern of lung micronodules, Mediastinal/pulmonary hilar nodes, CD4 T cell count, HIV viral load*.

## Discussion

The standard-of-care for the assessment of treatment success after ATT for pulmonary tuberculosis is sputum microscopy and culture. FDG PET/CT studies have reported residual or persisting metabolic activity in the lungs of patients declared cured based on the current standard of care (10, 11). We and others have previously shown that the presence of RMA in the lungs of patients declared cured according to the current standard of care is associated with early relapse of tuberculous disease (10, 11). These findings from previous studies suggest that FDG PET/CT may be a better modality for treatment response assessment than the current standard of care. Cost and availability are important issues that may limit the widespread application of FDG PET/CT for response assessment after ATT in routine care of patients with tuberculous disease. CT is a more readily available and cheaper modality in clinical practice. Therefore, in this current study, we sought to evaluate the strength of the associations of the different lung changes seen in patients who completed ATT and declared cured according to the current standard of care and the presence of RMA on FDG PET. We classified these CT changes into those known to be associated with active tuberculous disease and those associated with inactive lung changes induced by previous tuberculous disease. Out of the five CT features we classified as associated with active tuberculous disease, four showed a significant association with the presence of RMA on EOT FDG PET. The presence of pleural effusion was the only CT feature that did not show a significant association with the presence of RMA. This negative finding was understanding considering that pleural effusion was seen in only two patients in our study population. We also demonstrated a significant association between the combination of these five CT features of active tuberculous disease and the presence of RMA. The CT features we used as indicators of active tuberculous disease in our study have been previously reported as findings seen in patients starting ATT after the diagnosis of pulmonary tuberculosis (14–16). They have not been robustly assessed in patients who have completed ATT. Therefore, our findings are novel and may suggest that these known CT features of active tuberculous disease may be applied for treatment response assessment in lieu of FDG PET/CT in circumstances where the latter modality is not available or inaccessible.

The five CT features we classified as suggestive of inactive lung changes induced by successfully treated pulmonary tuberculosis disease, when applied in combination, showed no association with the presence of RMA on FDG PET, suggesting that the combination of these findings may help identify patients who have achieved a sterilizing cure. The individual CT features of inactive disease were not great in the assessment of their association with RMA. Two of the five CT features classified as suggestive of inactive disease showed significant associations with the presence of RMA. This finding is best understood on the background of the known heterogeneity of TB lesions where some lesions improved over time on treatment while some other lesions worsened or new lesions appearing in a patient on treatment (10, 18). For this reason, it is not uncommon to encounter, in the same patients, foci of inactive lung changes and other foci of active tuberculous disease. This heterogeneity in response to ATT that may be encountered in tuberculous disease is another strength of imaging for assessing the total-body burden of the disease and the response of each focus of disease to treatment. To address the heterogeneity of lung changes post treatment of pulmonary tuberculosis, a composite score derived from a combination of CT features may be better than considering each individual CT feature on their own. We showed that combined CT features of inactive disease showed a stronger strength of association with absence RMA on FDG PET than the individual CT feature of inactive disease on their own. Yen et al. in a cohort of 50 patients, derived a composite score for assessing lung changes seen in CT in the post tuberculosis setting (19). We did not apply the classification system utilized by Yen et al. because their study population is different from us. While we recruited patients who completed standard course of ATT and required no further treatment according to the current standard of care, Yen et al. studied patients with tuberculosis-induced destroyed lung syndrome for which the patients underwent surgical lung resection.

The greatest strength of FDG PET/CT in the management of patients with tuberculous disease is in its excellent negative predictive value, where a complete metabolic response to ATT is strongly associated with a sterilizing cure (11). On the contrary, not all patients with RMA eventually experience disease relapse on follow-up (11). RMA is primarily due to the persistence of dormant or slowly replicating but non-culturable bacilli that are difficult to eradicate with chemotherapy (6, 10, 20). The presence of these bacilli within tuberculous lesions stimulates host immune response giving rise to the FDG uptake in the lesions. Disease relapse due to the persisting bacilli occurs from the imbalance in the interplay between the host immune system and the bacterial virulence factors. A balance between these two factors (host and bacterial) ensures that the bacilli remain dormant without causing clinically overt disease. Another explanation for RMA that may be seen after the completion of ATT is the host inflammatory response induced by the lipid components of the dead bacterial cell membrane (21). FDG PET/CT was acquired within 2 weeks of completing ATT in our study. For this reason, a possibility exists that some of the patients with RMA in our study may have inflammation induced by dead bacilli rather than dormant or slowly replicating live bacilli. No FDG metric exists currently to differentiate between metabolic activity due to dead bacilli vs. activity due to live bacilli capable of causing disease relapse in the future. The discrimination between these two is of potential clinical importance and should be explored in future studies.

People living with HIV infection are at a higher risk of tuberculous disease, 10% risk per year in HIV-infected people compared with 10–15% lifetime risk in HIV-uninfected people (22, 23). This explains the prevalence of HIV infection seen in our study cohort, with two-thirds of them living with HIV infection at the time of this study. HIV is known to predispose to other inflammatory and infectious conditions, all of which may be confounders in the assessment of residual tuberculous lesion on FDG PET/CT after ATT (24). However, the presence of HIV was not significantly associated with the presence of RMA in our study cohort. This is not surprising considering that all HIV-infected patients included in our study were on stable antiretroviral treatment. This is in line with our experience where we have found the presence of HIV infection to not impact negatively on the accuracy of the quantification of the whole-body burden of disease in oncology patients (25–27). This observation is valid for HIV-infected patients on stable antiretroviral therapy. Interestingly, among HIV-infected patients, a CD4 T cell count of >200 cells/μL and a suppressed HIV viremia were significantly associated with the presence of RMA. These findings suggest that immune recovery evident by a high CD4 T cell count level is needed to mount a robust host immune response responsible for the RMA seen on EOT FDG PET. By extension, we speculate that advanced HIV infection may reduce RMA incidence regardless of the presence of persisting live bacilli.

The strengths of our study include its prospective design and the inclusion of a relatively large study population. At the same time, the study has got some important limitations. We did not acquire an FDG PET/CT scan at the commencement of ATT. Some of the lesions we classified as due to tuberculosis, especially those classified as suggestive of inactive post-tuberculous changes, could have predated the index tuberculous disease. We categorized cavitary lung lesions as suggestive of inactive tuberculous disease. In fact, lung cavity develops during the clinical stage of the disease. This may explain why the presence of cavitary lung lesion showed a significant association with the presence of RMA on EOT FDG PET in our analysis. Cavitary lesions are non-resolving so that they remain after a successful treatment regardless of when they develop. On this premise, we decided to classify cavitary lung lesions as representative of inactive lung changes induced by tuberculous disease rather than suggestive of active tuberculous disease. The classification of CT features as representing active disease was based on findings in the published literature (14–16). Unlike RMA on EOT FDG PET that has been robustly evaluated for its association with residual active tuberculosis, no such comprehensive assessment of the CT features characterized as representing active tuberculosis has been done in longitudinal studies. Our study did not assess the long-term outcome of patients with the CT features of active disease, representing a potential limitation that must be borne in mind when applying our study findings in clinical practice. Our study included mostly patients who completed a standard course of ATT for drug-sensitive TB. Drug-resistant tuberculosis presents a problem of larger magnitude in routine clinical practice as it is more difficult to treat, and the length of treatment is highly variable. It is unknown if the results from this study will be applicable in patients with drug-resistant tuberculosis.

## Conclusion

We showed that CT features such as lung nodules, lung consolidation, micronodules in the tree-in-bud pattern, and thoracic lymph nodes, either alone or in combination, have a significant association with residual metabolic activity on end-of-treatment FDG PET/CT in patients who completed a standard course of anti-tuberculous treatment. This suggests that FDG PET/CT may be substituted with CT in assessing the treatment outcome of patients with pulmonary tuberculosis due to its limited availability and high cost. HIV infection does not confound the interpretation of FDG PET/CT obtained from treatment response assessment of pulmonary tuberculosis.

## Data Availability Statement

The original contributions presented in the study are included in the article/supplementary material, further inquiries can be directed to the corresponding author/s.

## Ethics Statement

The studies involving human participants were reviewed and approved by Faculty of Health Sciences Research Ethics Committee, University of Pretoria. The patients/participants provided their written informed consent to participate in this study. Written informed consent was obtained from the individual(s) for the publication of any potentially identifiable images or data included in this article.

## Author Contributions

CH, JE, MH, BE, and MS designed the study. IL, KM, IM, NM, and GP collected and analyzed the data. MM, MN, BF, and MS supervised the project and supervision. IL write the original draft of the manuscript. All authors contributed to the article and approved the submitted version.

## Funding

The study received funding from CRDF Global for the project titled: The Clinical Research Unit (CRU) for the Advancement of Tuberculosis (TB) Biomarker-Targeted Interventions.

## Conflict of Interest

The authors declare that the research was conducted in the absence of any commercial or financial relationships that could be construed as a potential conflict of interest.

## Publisher's Note

All claims expressed in this article are solely those of the authors and do not necessarily represent those of their affiliated organizations, or those of the publisher, the editors and the reviewers. Any product that may be evaluated in this article, or claim that may be made by its manufacturer, is not guaranteed or endorsed by the publisher.
